# Predictors of the Atrial Fibrillation Following Catheter Ablation of Typical Atrial Flutter.

**DOI:** 10.5812/cardiovascmed.9061

**Published:** 2013-05-20

**Authors:** Majid Haghjoo, Nasim Salem, Masoud Rafati, Amirfarjam Fazelifar

**Affiliations:** 1 Cardiac Electrophysiology Research Center, Rajaie Cardiovascular Medical and Research Center, Tehran University of Medical Sciences, Tehran, IR Iran; 2 Rajaie Cardiovascular Medical and Research Center, Tehran University of Medical Sciences, Tehran, IR Iran

**Keywords:** Atrial Flutter, Ablation, Atrial Fibrillation

## Abstract

**Background:**

Despite technical refinements and improved long-term efficacy of the ablation procedure for treating AFL (AFL), the subsequent occurrence of AF (AF) following this procedure remains a significant clinical problem.

**Objectives:**

To determine long-term incidence and predictors of AF after catheter ablation of typical AFL.

**Material and Methods:**

Between March 2005 and February 2010, a total of 84 consecutive patients who underwent catheter ablation of documented typical AFL were enrolled.

**Results:**

Cavotricuspid isthmus ablation was successful in terminating and preventing the re-induction of AFL in all 84 patients (100%). The mean follow-up duration for study was 26± 22 months. During the follow-up period, early AF occurred in 5% after successful catheter ablation of AFL and late AF in 11% of the patients. The clinical variables associated with the occurrence of AF after catheter ablation of AFL were female, a history of AF before AFL ablation, body mass index (BMI), and left atrial abnormality. However, logistic multivariate analysis demonstrated that only BMI was independently associated with the late AF (OR 1.36, 95% CI 1.11-1.70, P = 0.004).

**Conclusions:**

Catheter ablation of flutter circuit will not prevent later manifestation of AF in 16% of the patients undergoing catheter ablation of the typical AFL. BMI was the only independent predictor of AF following catheter ablation of the typical AFL.

## 1. Background

Atrial flutter (AFL) has many similar clinical aspects to AF (AF) including underlying disease, predisposing factors, complications, as well as medical management approaches; the underlying mechanism of AFL makes it amenable to cure with catheter-based techniques (1-7). The prognosis of ablation for patients with type I AFL who undergo this caterer catheter is excellent, with a very low recurrence rate however, the picture is not as clear for patients with concomitant AFL and AF (8). Some reports have documented fewer episodes of AF after successful flutter ablation, while others have not (9-11). Despite technical refinements and improved long-term efficacy of the ablation procedure, the subsequent occurrence of AF remains a significant clinical problem. Indeed, previous studies reported a 10% to 61% incidence of AF after ablation procedures (10-12). However, there are limited information regarding the frequency, duration, and therapy of AF in individual patients.

## 2. Objectives

We sought to determine the long-term incidence and the predictors of AF following catheter ablation of typical AFL.

## 3. Material and Methods

### 3.1. Study Population and Demographics

The study population consisted of 84 consecutive patients (63 men, mean age of 49 ± 17 years) who underwent catheter ablation of typical documented AFL between March 2005 and February 2010 in our center. The predominant clinical arrhythmia was typical AFL in all cases. However, in some patients, AF had been documented or suspected before ablation. The clinical variables analyzed in relation to the later occurrence of AF were the duration of AFL before ablation, the left ventricular (LV) ejection fraction, sex, age, body mass index (BMI), prior history of documented AF, the presence and type of structural heart disease, the inducibility of sustained AF after ablation of AFL, and etc.

### 3.2. Electrophysiologic Study and Radiofrequency Ablation

All patients provided written informed consent for electrophysiologic study and radiofrequency (RF) ablation. The study protocol was approved by the Review Board for Human Subjects of the Tehran University of Medical Sciences. The patients were studied in postabsorptive state and after all antiarrhythmics except for those intending to slow atrioventricular nodal conduction that had been discontinued for at least 5 half-lives. 

Electrophysiologic study was performed using standard methods. Briefly, one 6F quadripolar catheter was introduced through the left femoral vein and was advanced under fluoroscopic guidance to the right ventricular apex. In all patients undergoing the standard procedure, a 7F decapolar catheter was positioned retrogradely via the left femoral vein into the coronary sinus and another 7F dodecapolar catheter was placed within the right atrium via right femoral vein. After confirming cavotricuspid isthmus (CTI) dependency of the AFL, linear RF lesions were applied throughout the CTI starting from ventricular side of the tricuspid annulus and ending within the junction of CTI and inferior vena cava. In all patients, RF energy was delivered using irrigated catheter and long steerable sheath during spontaneous or inducible AFL, except for the patients in whom no AFL was inducible. The endpoint of ablation was bidirectional block across the CTI persisting after a 30-minute waiting period.

### 3.3. Follow-up

All patients were visited one month after catheter ablation and every 3-month then after. The incomplete data were followed up by telephone contact. No antiarrhythmics were administered after ablation; however, warfarin was prescribed in all patients at least for 6 to 8-week. Arrhythmia recurrence was detected using 12-lead ECG taken on follow-up visit and ambulatory holter monitoring in case of palpitations.

### 3.4. Statistical Analysis

The data were recorded in SPSS 17 for windows (SPSS Inc. Chicago, IL, USA). Continuous variables were expressed as mean ± SD and categorical variables as number and percentage (%). Univariate analysis of factors associated with the late occurrence of AF was performed using the Student's t test for continuous and chi-square test or Fisher's exact test for categorical variables. A value of P ≤.05 was considered to indicate statistical significance. Stepwise multivariate logistic regression analysis was performed to determine the independent predictors of the late development of AF, with all variables having a univariate association at a significance level of ≤ 0.1 included in the model.

## 4. Results

### 4.1. Study Population

The clinical characteristics of the study population are summarized in Table 1. There were 63 men and 21 women, with a mean age of 49 ± 17 years and mean BMI of 26 ± 4.5 kg/m2. All patients were refractory to at least one class 1 or 3 antiarrhythmic drug. The mean duration of symptoms before referral for ablation was 20 ± 4 months. AFL was paroxysmal in 55% of the patients and the rest of the patients had persistent AFL. The mean AFL cycle length was 288 ± 98 msec. The most common clinical symptoms were palpitation (88%), followed by dyspnea (5%), syncope (4%), and dizziness (2%). Regarding underlying cardiac abnormalities, congenital heart disease was present in 21%, coronary artery disease in 18%, valvular disease in 9.5%, dilated cardiomyopathy in 7%, and hypertrophic cardiomyopathy in 1%. Systolic hypertension was prevalent in 18%, diabetes mellitus in 8%, and smoking in 17%. Mean left ventricular ejection fraction was 44 ± 12%, mean left atrial dimension was 38 ± 8 mm, and LV end-diastolic and end-systolic dimensions were 49 ± 10 mm and 38 ± 10 mm, respectively. Fifty-six patients (67%) had evidences of mitral regurgitation (MR); most cases had mild MR. Left or right atrial abnormalities were found in 30% and 9.5%, respectively (Table 2). Discharge medications consisted of beta-blockers in 40%, statins in 20%, and angiotensin-converting enzyme inhibitors in 34%.

**Table 1.** Baseline Demographic and Clinical Characteristics

	Values^a^
**Age (yrs)**	49 ± 17
**Male**	63 (75)
**Body mass index,kg/m^2^**	26 ± 4.5
**Symptom duration (months)**	20 ± 4
**Clinical presentation**	
Palpitation	74 (88)
Dyspnea	4 (5)
Syncope	3 (4)
Dizziness	2 (2)
Asymptomatic	1 (1)
**Preablationcerebrovascular accident**	1 (1)
**Chronic obstructive pulmonary disease**	3 (4)
**Systolic hypertension**	15 (18)
**Diabetes mellitus**	7 (8)
**Cigarette smoking**	14 (17)
**Preablationatrial fibrillation**	11 (13)
**Underlying heart disease:**	
Congenital heart disease	18 (21)
Coronary artery disease	15 (18)
Valvularheart disease	8 (9.5)
Dilated cardiomyopathy	6 (7)
Hypertrophic cardiomyopathy	1 (1)
**Pre-ablation flutter type**	
Paroxysmal	46 (55)
Persistent	38 (45)

^a^ All values are in mean ± SD

**Table 2.** Baseline Echocardiographic Characteristics

	Values^a^
**LV**^b^ejection fraction(%)	44 ± 12
**LA**^b^diameter(mm)	38 ± 8
**LA appendage velocity(cm/s)**	46 ± 24
**LV end-diastolic diameter(mm)**	49 ± 10
**LV end-systolic diameter(mm)**	38 ± 10
**E wave**	97 ± 44
**A wave**	87 ± 15
**P wave**	83 ± 19
**LA abnormality**	25 (30)
**RA**^b^abnormality	8 (9.5)
**Mitralregurgitation**	
Mild	51 (61)
Moderate	4 (5)
Severe	1 (1)

^a^ All values are in mean ± SD

^b^ Abbreviations: LV, Left Ventricular; LA, Left Atrial; RA, Right Atrial

### 4.2. Catheter Ablation

First, radiofrequency catheter ablation was successful in terminating and preventing the re-induction of AFL in 74 of 84 patients (88%). Remaining 10 (22%) patients required second procedures for successful catheter ablation of AFL. Inducible AF was observed in 15%. The mean follow-up duration was 26 ± 22 months. No major complications were observed during catheter ablation or before the discharge.

### 4.3. Follow-up Data

Recurrence of AFL was documented in 13.0% of patients during follow-up. AF occurred in 5% of the patients within the first month following successful catheter ablation and in 11% after the first month. The clinical variables associated with the occurrence of AF after RF catheter ablation of AFL in univariate analysis (Table 3) were:

1. female gender,
2. a history of AF before AFL ablation,
3. BMI, and
4. left atrial abnormality.

In addition, there was a trend for higher incidence of AF in patients with inducible AF and those who did not receive angiotensin-converting enzyme inhibitors after discharge. However, stepwise multivariate analysis demonstrated that only BMI was independently associated with the late development of AF (OR 1.36, 95% CI 1.11-1.70, P = 0.004).

**Table 3.** Clinical, Echocardiographic, Ablation and Follow-up data in Patients With and Without AF After Catheter Ablation

	With AF^a^(N =9)	Without AF^a^(N = 75)	P value
**Female gender**	5 (56)	16 (21)	0.02
**Age(yrs)**	51 ± 20	49 ± 17	0.82
**Body mass index (kg/m^2^)**	30 ± 6	25 ± 4	0.02
**Symptom duration (month)**	22 ± 4	20 ± 4	0.90
**Clinical symptoms**			0.57
Palpitation	7 (78)	67 (89)	
Dyspnea	1 (11)	3 (4)	
Syncope	1 (11)	2 (3)	
Dizziness	0	2 (3)	
Asymptomatic	0	1 (1)	
**History of cerebrovascular accident**	0	1 (1)	0.73
**History of chronic obstructive pulmonary disease**	1 (11)	2 (3)	0.20
**Systolic hypertension**	3 (33)	12 (16)	0.20
**Diabetes mellitus**	0	7 (9)	0.34
**Cigarette smoking**	1 (11)	13 (17)	0.64
**Pre-ablation atrial fibrillation**	3 (33)	8 (11)	0.05
**Underlying heart disease**			0.52
Congenital	3 (33)	15 (20)	
Coronary	0	15 (20)	
Valvular	0	8 (11)	
Dilated cardiomyopathy	1 (11)	5 (7)	
Hypertrophic cardiomyopathy	0	1 (1)	
**Pre-ablation flutter type**			0.65
Counterclockwise	7 (78)	53 (71)	
Clockwise	2 (22)	22 (29)	
**Mitral regurgitation**	8 (89)	59 (79)	0.47
**Left ventricular hypertrophy**	0	8 (11)	0.30
**Left ventricular ejection fraction(%)**	42 ± 12	44 ± 12	0.79
**Left atrial abnormality**	0	25 (33)	0.04
**Right atrial abnormality**	0	8 (11)	0.30
**Inducible atrial fibrillation**	3 (33)	10 (13)	0.11
**Postdischargestatin**	1 (11)	16 (21)	0.47
**Postdischargeangiotensin converting enzyme inhibitor**	1 (11)	28 (37)	0.11

^a^ All values are in mean ± SD or n (%)

## 5. Discussion

The major findings of the study were as follows:

1. AF occurred in 16% of the patients undergoing cavotricuspid ablation for typical AFL;
2. BMI was the only independent predictor of AF following catheter ablation of cavotricuspid isthmus.

During mean follow-up duration of 26 months, we observed an AF incidence of 16% following typical AFL ablation. Our data was in agreement with previous publications. In a similar study by Laurent et al(13) on 148 consecutive patients who underwent cavotricuspid isthmus ablation for the treatment of typical AFL, AF occurred in 27% of the patients during an average follow-up of 21 months. In the study by Da Costa et al, AF was documented in 16 (17%) of the 96 consecutive ablated patients 30 days after ablation (14). Tai et al. showed that 21.5% of patients undergoing AFL ablation developed AF during the follow-up period (15). In addition, Paydak and his colleagues (16) revealed that among 110 consecutive patients with ablation of typical AFL, AF was documented in 25% during a mean follow-up of 20 months. Moreover, Philippon et al. (8) observed that AF occurred in 14 of 53 patients after successful ablation (26.4%). Generally, it can be concluded that AF occurred in a significant percentage (16%-25%) of the patients undergoing cavotricuspid ablation for typical AFL.

Therefore, it appears that these patients need close monitoring after ablation to detect recurrent AF and administer appropriate anticoagulant and/or antiarrhythmic medications.We also observed that female gender, history of preablation AF, the presence of identifiable structural heart disease, BMI, and left atrial abnormality were associated with higher risk of AF occurrence after AFL ablation. However, in our multivariable model, BMI was the only main independent predicting factor. With respect to predictors of AF in other studies, history of AF before ablation, structural heart defects, or even some medications have been reported to predict this event. In Laurent’s study, only inducible AF and paroxysmal AFL were independent factors linked to AF after ablation (13). In Da Costa’s study, multivariate analysis using a Cox model showed that the only independent predictors of early AF were left ventricular ejection fraction and pre-ablation history of AF (14). Tai et al. found that history of AF and inducible sustained AF could predict the late development of AF after AFL ablation (15). Furthermore, Philippon showed that only the persistent inducibility of sustained AF predicted the later development of AF (8). With respect to the relationship between obesity and higher incidence of AF, some authors showed that BMI is a strong predictor for future development of AF and should be considered as a risk factor for AF (17, 18). However, a few studies have focused on the role of obesity in higher occurrence of AF, especially following ablation that needs to be more investigated in further studies. 

### 5.1. Limitations

The results of this study should be interpreted in the light of certain limitations. First, our study is limited by its retrospective analysis. Second, we may also have underestimated the patients with asymptomatic episodes of AF, because we routinely used only 12-lead ECG and ambulatory ECG monitoring for the selected patients. 

Eradication of the atrial flutter circuit will not prevent later manifestation of AF in a significant number of the patients. Body mass index was the only predictor for AF following catheter ablation of the typical AFL.
